# Comparison of Non-contact Tonometry and Goldmann Applanation Tonometry Measurements in Non-pathologic High Myopia

**DOI:** 10.3389/fmed.2022.819715

**Published:** 2022-03-03

**Authors:** Peiyuan Wang, Yunhe Song, Fengbin Lin, Zhenyu Wang, Xinbo Gao, Weijing Cheng, Meiling Chen, Yuying Peng, Yuhong Liu, Xiulan Zhang, Shida Chen

**Affiliations:** State Key Laboratory of Ophthalmology, Zhongshan Ophthalmic Center, Sun Yat-sen University, Guangdong Provincial Key Laboratory of Ophthalmology and Visual Science, Guangdong Provincial Clinical Research Center for Ocular Diseases, Guangzhou, China

**Keywords:** intraocular pressure, high myopia, non-contact tonometry, Goldmann applanation tonometry, axial length, central corneal thickness, corneal curvature

## Abstract

**Purpose:**

To compare intraocular pressure (IOP) values obtained using Goldmann applanation tonometry (IOP_GAT_) and non-contact tonometry (IOP_NCT_) in a non-pathologic high myopia population.

**Methods:**

A total of 720 eyes from 720 Chinese adults with non-pathologic high myopia were enrolled in this cross-sectional study. Demographic and ocular characteristics, including axial length, refractive error, central corneal thickness (CCT), and corneal curvature (CC) were recorded. Each patient was successively treated with IOP_NCT_ and IOP_GAT_. Univariate and multivariable linear regression analyses were conducted to detect factors associated with IOP_NCT_ and IOP_GAT_, as well as the measurement difference between the two devices (IOP_NCT−GAT_).

**Results:**

In this non-pathologic high myopia population, the mean IOP_NCT_ and IOP_GAT_ values were 17.60 ± 2.76 mmHg and 13.85 ± 2.43 mmHg, respectively. The IOP measurements of the two devices were significantly correlated (*r* = 0.681, *P* < 0.001), however, IOP_NCT_ overestimated IOP_GAT_ with a mean difference of 3.75 mmHg (95% confidence interval: 3.60–3.91 mmHg). In multivariate regression, IOP_NCT_ was significantly associated with body mass index (standardized β = 0.075, *p* = 0.033), systolic blood pressure (SBP) (standardized β = 0.170, *p* < 0.001), and CCT (standardized β = 0.526, *p* < 0.001). As for IOP_GAT_, only SBP (standardized β = 0.162, *p* < 0.001), CCT (standardized β = 0.259, *p* < 0.001), and CC (standardized β = 0.156, *p* < 0.001) were significantly correlated. The mean IOP_NCT−GAT_ difference increased with younger age (standardized β = −0.134, *p* < 0.001), higher body mass index (standardized β = 0.091, *p* = 0.009), higher SBP (standardized β = 0.074, *p* = 0.027), thicker CCT (standardized β = 0.506, *p* < 0.001), and lower IOP_GAT_ (standardized β = −0.409, *p* < 0.001).

**Conclusion:**

In the non-pathologic high myopia population, IOP_NCT_ overestimated IOP_GAT_ at 3.75 ± 2.10 mmHg. This study suggests that the difference between the values obtained by the two devices, and their respective influencing factors, should be considered in the clinical evaluation and management of highly myopic populations.

## Introduction

High myopia is an extreme form of myopia, mainly characterized by excessive axial elongation and various pathological ocular lesions (1). A growing body of evidence suggests that high myopia is closely related to the occurrence of glaucoma (2–7). As intraocular pressure (IOP) is the most important target and the only treatable factor in glaucoma, accurate and reliable measurement as well as monitoring of IOP are essential in highly myopic populations.

Many types of devices have been proposed for IOP measurement, such as Goldmann applanation tonometry (GAT), non-contact tonometry (NCT), ICare rebound tonometer, and dynamic contour tonometer, each of which has advantages and disadvantages (8). The GAT is widely regarded as the gold standard for IOP measurement due to its accuracy and excellent reproducibility, while NCT is most widely used in outpatients and in ocular hypertension screening because of its non-invasive and convenient nature (9, 10). However, all measurements are influenced by the structure and biomechanical properties of the cornea, such as the cornea thickness and hysteresis, as well as systemic factors such as systolic pressure (11–13).

Due to excessive elongation of axial length (AL) in high myopia, which is accompanied by changes in scleral and corneal structures and their biomechanics, IOP values obtained may vary among different tonometers. Previous studies have reported the distribution of IOP values in high myopia populations, ranging from 9 to 27 mmHg, as measured by the GAT or NCT (14, 15). Comparative studies on IOP measurement with the NCT and GAT in high myopia populations are limited, and results from different measurements may not be comparable.

The present study aimed to evaluate the difference in IOP measurements obtained with the NCT and GAT in a population with non-pathologic high myopia, and the demographic and ocular characteristics that affect these measurements. The present findings may inform a more comprehensive and reliable management of IOP in highly myopic patients.

## Materials and Methods

### Study Participants

This prospective cross-sectional study was conducted at the Zhongshan Ophthalmic Center (ZOC), Sun Yat-sen University, Guangzhou. Participants were recruited from a registry cohort study on the natural history of myopic neuropathy, which started from June 2019 to June 2021 (ClinicalTrials.gov identifier: NCT04302220) (16). This study was approved by the Ethics Review Committee of the ZOC, and the study procedure conformed to the Declaration of Helsinki. Informed consent was obtained from all the participants prior to enrollment.

High myopia adults aged 18–65 years were recruited, as previously reported (16). Briefly, patients were eligible for this study if either the left or right eye presented with myopic spherical equivalence (SE) of ≤ -6 diopters or AL of ≥26.5 mm, best corrected visual acuity (BCVA) of ≥6/12, and myopic maculopathy category 0 or 1 [based on the International Photographic Classification and Grading System for Myopic Maculopathy (17)]. The right eye was chosen for analysis if both eyes met the inclusion criteria.

The exclusion criteria were as follows: (1) patients with severe systemic diseases such as malignant tumors, (2) a history of ocular surgery or laser treatment, and (3) ocular infection diseases that could not be measured by the GAT, such as corneal ulcers.

### Demographic and Ocular Characteristics

In this study, blood pressure was measured twice using an automated blood pressure apparatus (Omron Healthcare Ltd., Japan), and the average value was recorded. Body mass index (BMI) was calculated as body weight (kg) divided by height (m) squared. Demographic and clinical characteristics such as age, sex, and other medical history data were collected through interviews.

All subjects underwent a comprehensive ophthalmic examination at the ZOC Clinical Research Center, including BCVA assessment, refractive error assessment with an autorefractor (KR-800, Topcon, Japan); slit lamp bio-microscopy (BQ-900, Haag-Streit, Switzerland), IOP measurement by both the NCT (mputeCT-1 Corized Tonometer, Topcon Ltd., Topcon) and GAT (Haag-Streit, Koniz, Switzerland); AL, central corneal thickness (CCT), and corneal curvature (CC) values were obtained using an IOL Master (IOL Master 700, Carl Zeiss Meditec, Germany); digital stereo fundus photography (Nonmyd WX3D, KOWA, Japan) was performed. All measurements were performed by well-trained technicians.

### IOP Measurement

IOP was measured in all participants between 9:00 a.m. and 11:00 a.m. to minimize the influence of IOP circadian variations. Each participant was asked to calm down for at least 5 min before the measurement. The NCT assessment was performed 15 min before the GAT assessment; the assessments were performed three times at 1-min intervals. Both instruments were periodically calibrated according to the manufacturer's guidelines, and the operations were conducted strictly following the manufacturer's instructions. During the GAT measurement, 0.5% proparacaine hydrochloride eye drops (Alcon, Fort Worth, TX, USA) were used for topical anesthesia, and fluorescein strips (Liaoning Meizilin Pharmaceutical Co., Ltd., China) were gently applied to the palpebral conjunctiva for corneal staining. The IOP measurement with the GAT was performed using a slit lamp mounted applanation tonometer and performed by the same experienced ophthalmologist twice, and the average values were recorded.

### Statistical Analysis

In descriptive analysis, continuous variables were summarized as mean ± standard deviation and range, and categorical variables were presented as frequency and proportion. The Bland-Altman analysis was performed to evaluate the agreement between IOP_NCT_ and IOP_GAT_ (18). The mean difference and limits of agreement (LOA) were calculated to quantify the extent of bias between the two measurements. Pearson's coefficient was used to assess the correlation between IOP_NCT_ and IOP_GAT_. Linear regression analyses were conducted to determine the factors associated with IOP_NCT_, IOP_GAT_, and IOP_NCT−GAT_. Variables with *p*-values of <0.1 in univariable linear regression were included in multivariable linear regression. All multivariable models were adjusted for age. Statistical significance was set at *p*-values of <0.05. Statistical analyses were performed using Stata 16 software (Stata Corp., T.X., USA).

## Results

### Participants' Characteristics

A total of 812 subjects were enrolled in this study; subsequently, 22 patients with a history of ocular surgery or laser treatment, 10 patients with ocular trauma or infection that could not complete a GAT measurement, 9 patients with severe systemic diseases, 34 patients using ocular hypotensive agents, and 17 patients with missing values of SE or IOL master measurement were excluded. Finally, a total of 720 subjects (720 eyes) with non-pathologic high myopia were included in the analysis. Table 1 presents the clinical characteristics of the participants. The mean age was 31.10 ± 9.86 years (range 18 to 64 years), and 271 (37.64%) participants were male. The average AL, SE, CCT, and CC values were 26.90 ± 1.19 mm,−8.85 ± 2.22 diopter, 540.54 ± 32.03 μm, and 43.80 ± 1.44 diopter, respectively.

**Table 1:** Demographic and ocular characteristics of the high myopia participants.

**Parameters**	**Mean ±SD**	**Range**
**Systemic-related parameters**		
Age (years)	31.10 ± 9.86	18–64
Sex, male/female	271/449	
BMI (kg/m^2^)	21.37 ± 3.09	15.42–37.64
SBP (mmHg)	114.05 ± 13.90	82–158
DBP (mmHg)	66.10 ± 10.14	40–119
**Ocular-related parameters**		
Axial length (mm)	26.90 ± 1.19	23.60–33.54
Central corneal thickness (μm)	540.54 ± 32.03	448.86–653.46
Corneal curvature (D)	43.80 ± 1.44	39.51–49.05
Spherical equivalence (D)	−8.85 ± 2.22	−20.13 to −6.00
**IOP (mmHg)**		
IOP_NCT_	17.60 ± 2.76	10–27
IOP_GAT_	13.85 ± 2.43	8–24

*Data are presented as mean ± standard deviation and range unless stated otherwise*.

*BMI, body mass index; D, diopter; DBP, diastolic blood pressure; IOP, intraocular pressure; IOP_NCT_, intraocular pressure measured with non-contact tonometer; IOP_GAT_, intraocular pressure measured with Goldmann applanation tonometer; SBP, systolic blood pressure; SD, standard deviation*.

### Difference Between IOP_NCT_ and IOP_GAT_ Measurements

**Figure 1:**
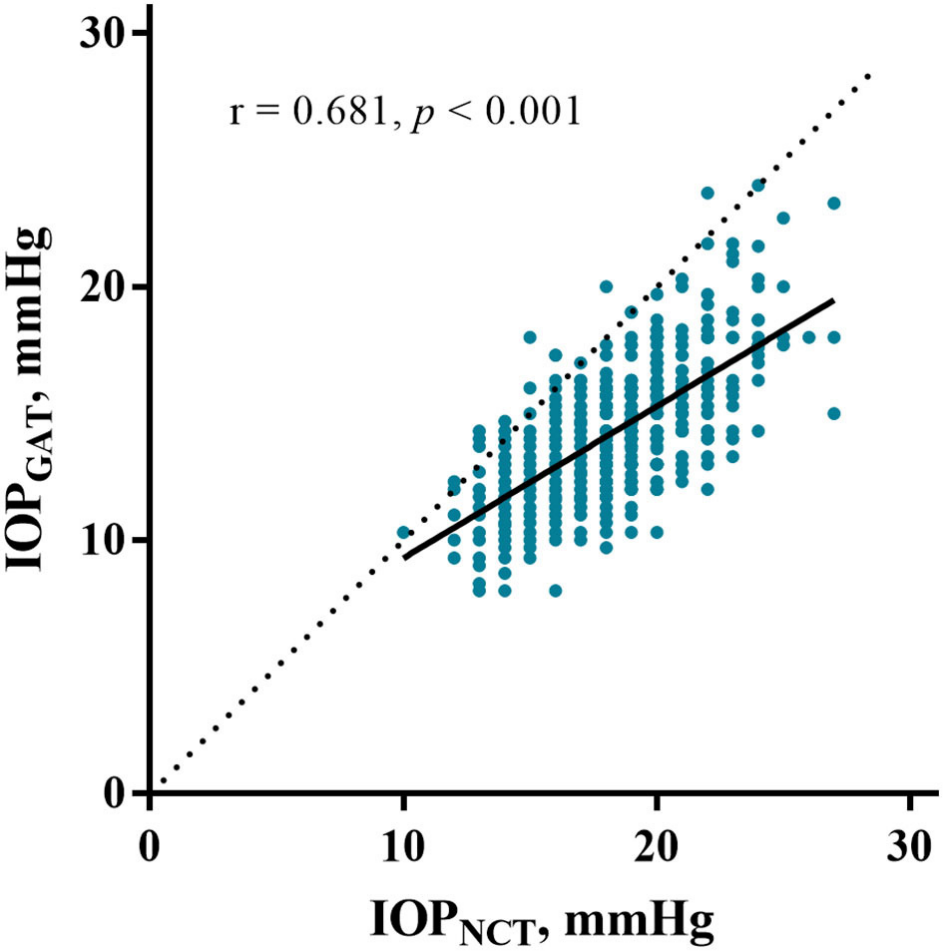
Correlation between IOP_NCT_ and IOP_GAT_. The scatter plot and regression line (solid line) show comparisons between IOP_NCT_ and IOP_GAT_ values in eyes with high myopia. The dotted line represents the line of identity, and r indicates Pearson's correlation coefficient. IOP_NCT_, intraocular pressure measured with non-contact tonometer; IOP_GAT_, intraocular pressure measured with Goldmann applanation tonometer.

**Figure 2:**
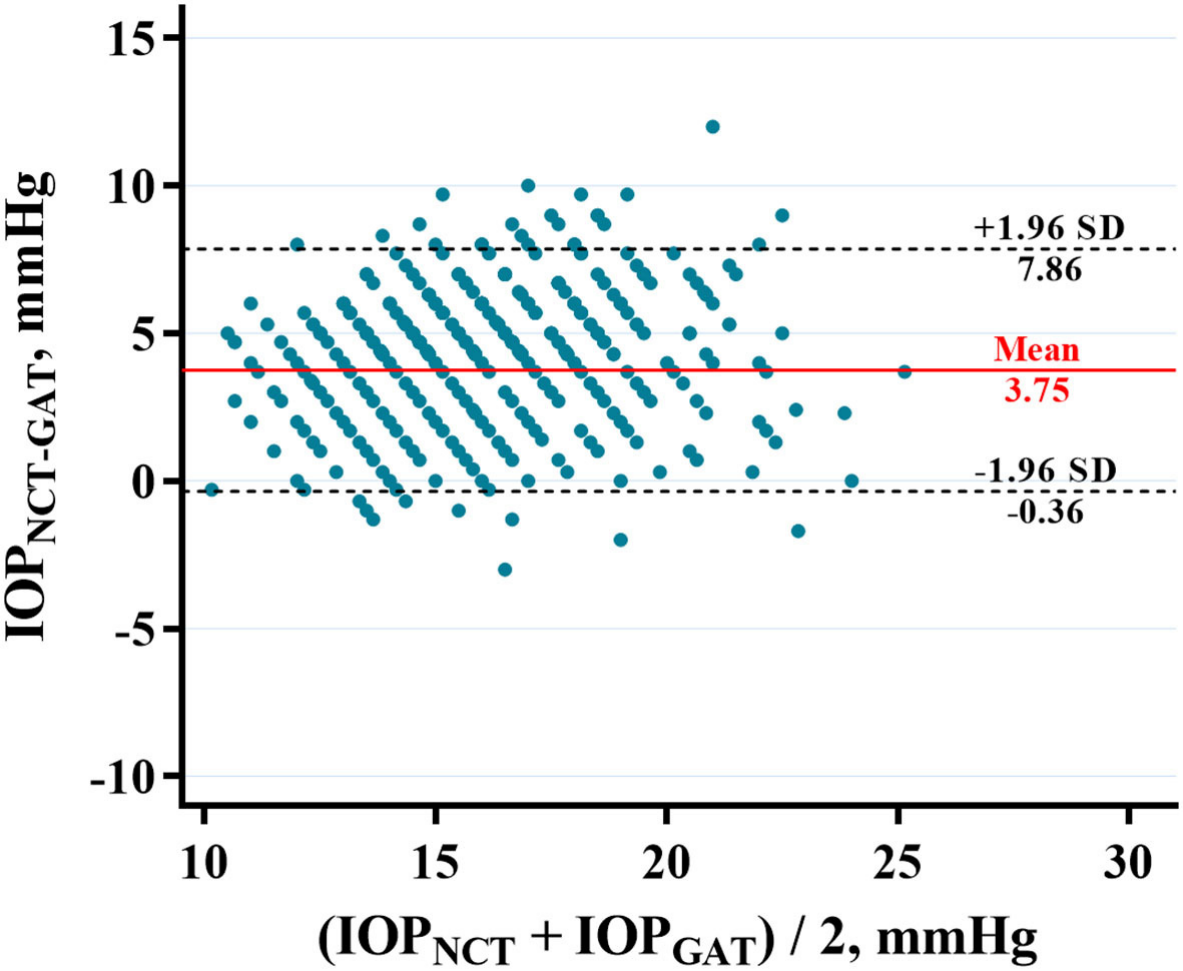
Bland-Altman plot showing the agreement between IOP_NCT_ and IOP_GAT_ in highly myopic eyes. X axis, mean of IOP_NCT_ and IOP_GAT_ values. Y axis, the difference between IOP_NCT_ and IOP_GAT_ values. The two black dashed lines indicate mean difference ± 1.96 standard deviation between IOP_NCT_ and IOP_GAT_ (−0.36–7.86 mmHg). The red line indicates the mean difference in IOP (3.75 mmHg). IOP_NCT_, intraocular pressure measured with non-contact tonometer; IOP_GAT_, intraocular pressure measured with Goldmann applanation tonometer.

The average IOP_NCT_ and IOP_GAT_ values were 17.60 ± 2.76 mmHg and 13.85 ± 2.43 mmHg, respectively (Table 1). IOP_NCT_ and IOP_GAT_ values were significantly correlated (*r* = 0.681, *P* < 0.001) in linear regression analysis (Figure 1). The Bland-Altman scatter plot showed that the mean difference between the two measurements was 3.75 mmHg, with LOA in the range of −0.35 to 7.86 mmHg. Only 5.14% (37/720) of IOP_NCT−GAT_ data points fell outside the LOA range (Figure 2).

### Factors Associated With IOP_NCT_ and IOP_GAT_

**Figure 3:**
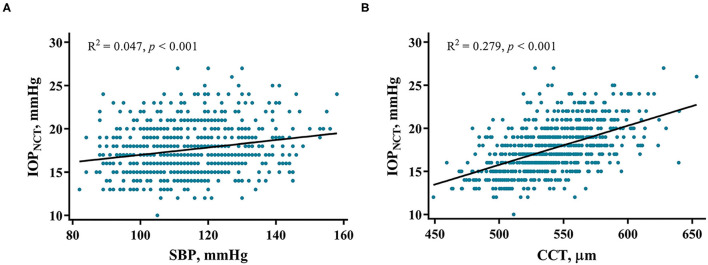
Correlations between IOP_NCT_ values and risk factors. Regression plots show systolic blood pressure (SBP) **(A)** and central corneal thickness (CCT) **(B)** effect on IOP_NCT_ with *R*^2^ = 0.047 and 0.279, respectively. The full lines indicate regression lines. IOP_NCT_, intraocular pressure measured with non-contact tonometer.

Univariate linear regression revealed that IOP_NCT_ was strongly associated with sex, BMI, SBP, and CCT. Multivariable regression analysis, which included IOP_NCT_ as the dependent variable, and age, sex, BMI, SBP, AL, and CCT as independent variables, showed that age (standardized β = −0.115, *p* < 0.001) was negatively associated with IOP_NCT_, while BMI (standardized β = 0.075, *p* = 0.033), SBP (standardized β = 0.170, *p* < 0.001), and CCT (standardized β = 0.526, *p* < 0.001) were positively correlated with IOP_NCT_ (Table 2). The related regression plots (Figure 3) showed that the *R*^2^ values of SBP and CCT on IOP_NCT_ were 0.047 and 0.279, respectively.

**Table 2:** Univariate and multivariable linear regression analyses of factors that affect IOP_NCT_.

	**Univariable linear regression**			**Multivariable linear regression**		
	***B*** **(95% CI)**	**β (95% CI)**	***p*** **-value**	***B*** **(95% CI)**	**β (95% CI)**	***p*** **-value**
Age, years	−0.006 (−0.027, 0.014)	−0.022 (−0.095, 0.051)	0.555	−0.032 (−0.050, −0.014)	−0.115 (−0.180, −0.051)	**<0.001**
Sex	−0.718 (−1.132, −0.304)	−0.126 (−0.199, −0.053)	**0.001**	0.177 (−0.231, 0.586)	0.031 (−0.041, 0.103)	0.394
BMI (kg/m^2^)	0.121 (0.056, 0.185)	0.135 (0.062, 0.208)	**<0.001**	0.067 (0.006, 0.128)	0.075 (0.006, 0.143)	**0.033**
SBP (mmHg)	0.043 (0.028, 0.057)	0.214 (0.142, 0.286)	**<0.001**	0.034 (0.020, 0.048)	0.170 (0.098, 0.242)	**<0.001**
DBP (mmHg)	0.061 (0.041, 0.080)	0.223 (0.152, 0.295)	**<0.001**			
AL (mm)	0.169 (−0.001, 0.338)	0.073 (−0.001, 0.146)	0.052	−0.040 (−0.188, 0.108)	−0.017 (−0.081, 0.047)	0.596
SE (D)	−0.086 (−0.177, 0.005)	−0.069 (−0.142, 0.004)	0.064			
CCT (μm)	0.046 (0.040, 0.051)	0.528 (0.466, 0.590)	**<0.001**	0.045 (0.040, 0.051)	0.526 (0.464, 0.588)	**<0.001**
CC (D)	−0.050 (−0.191, 0.091)	−0.026 (−0.099, 0.047)	0.487			

*SBP and DBP, Al and SE were correlated; therefore, only SBP and AL were included in multivariable regression*.

*Boldface values indicate statistical significance*.

*AL, axial length; B, non-standardized beta; BMI, body mass index; β, standardized beta; CC, corneal curvature; CCT, central corneal thickness; CI, confidence interval; D, diopter; DBP, diastolic blood pressure; GAT, Goldmann applanation tonometer; IOP_NCT_, intraocular pressure measured using a non-contact tonometer; SBP, systolic blood pressure; SD, standard deviation; SE, spherical equivalence*.

**Figure 4:**
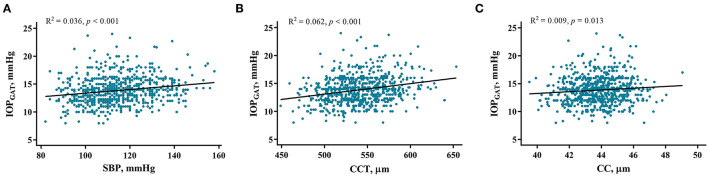
Correlations between IOP_GAT_ values and risk factors. Regression plots show systolic blood pressure (SBP) **(A)**, central corneal thickness (CCT) **(B)**, and corneal curvature (CC) **(C)** effect on IOP_GAT_ with *R*^2^ = 0.036, 0.062, and 0.009, respectively. The full lines indicate regression lines. IOP_GAT_, intraocular pressure measured with Goldmann applanation tonometer.

Univariate linear regression showed that IOP_GAT_ was significantly positively associated with sex, BMI, SBP, CCT, and CC (Table 3). Multivariable regression confirmed the results of univariate linear regression and revealed that only SBP (standardized β = 0.162, *p* < 0.001), CCT (standardized β = 0.259, *p* < 0.001), and CC (standardized β = 0.156, *p* < 0.001) were significantly associated with IOP_GAT_. However, age, sex, and BMI were not associated with the IOP_GAT_. The related regression plots (Figure 4) showed that the *R*^2^ values of SBP, CCT, and CC on GAT were 0.036, 0.062, and 0.009, respectively.

**Table 3:** Univariate and multivariable linear regression analyses of factors that affect IOP_GAT_.

	**Univariable linear regression**			**Multivariable linear regression**		
	***B*** **(95% CI)**	**β (95% CI)**	***p*** **-value**	***B*** **(95% CI)**	**β (95% CI)**	***p*** **-value**
Age, years	0.003 (−0.015, 0.022)	0.014 (−0.059, 0.087)	0.709	−0.011 (−0.030, 0.007)	−0.046 (−0.120, 0.028)	0.223
Sex	−0.577 (−0.942, −0.212)	−0.115 (−0.188, −0.042)	**0.002**	−0.162 (−0.565, 0.241)	−0.032 (−0.113, 0.048)	0.429
BMI (kg/m^2^)	0.058 (0.001, 0.115)	0.074 (0.001, 0.147)	**0.048**	0.014 (−0.048, 0.076)	0.018 (−0.061, 0.096)	0.657
SBP (mmHg)	0.033 (0.021, 0.046)	0.190 (0.118, 0.262)	**<0.001**	0.028 (0.014, 0.043)	0.162 (0.080, 0.244)	**<0.001**
DBP (mmHg)	0.042 (0.025, 0.060)	0.176 (0.104, 0.248)	**<0.001**			
AL (mm)	−0.062 (−0.212, 0.088)	−0.030 (−0.104, 0.043)	0.416			
SE (D)	−0.028 (−0.108, 0.052)	−0.025 (−0.099, 0.048)	0.496			
CCT (μm)	0.019 (0.014, 0.024)	0.249 (0.178, 0.320)	**<0.001**	0.020 (0.014, 0.025)	0.259 (0.188, 0.330)	**<0.001**
CC (D)	0.156 (0.033, 0.280)	0.092 (0.019, 0.165)	**0.013**	0.265 (0.143, 0.386)	0.156 (0.084, 0.228)	**<0.001**

*SBP and DBP were correlated; therefore, only SBP was included in multivariable regression*.

*Boldface values indicate statistical significance*.

*AL, axial length; B, non-standardized beta; BMI, body mass index; β, standardized beta; CC, corneal curvature; CCT, central corneal thickness; CI, confidence interval; D, diopter; DBP, diastolic blood pressure; IOP_GAT_, intraocular pressure measured with Goldmann applanation tonometer; SBP, systolic blood pressure; SD, standard deviation; SE, spherical equivalence*.

### Factors Affecting IOP_NCT-GAT_

**Figure 5:**
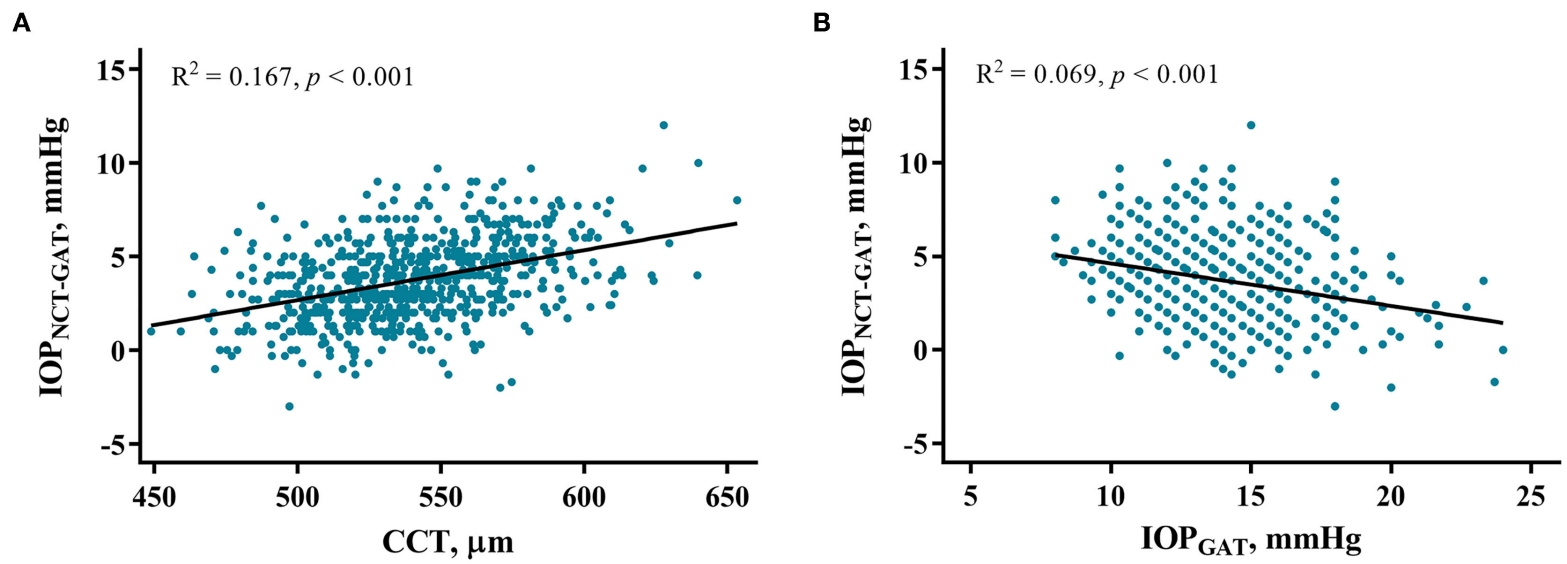
Correlations between IOP_NCT−GAT_ values and risk factors. Regression plots show central corneal thickness (CCT) **(A)** and IOP_GAT_ value **(B)** effect on IOP_NCT−GAT_ with *R*^2^ = 0.167 and 0.069, respectively. The full lines indicate regression lines. IOP_GAT_, intraocular pressure measured with Goldmann applanation tonometer; IOP_NCT−GAT_, the difference of intraocular pressure measured with non-contact and Goldmann applanation tonometer.

IOP_NCT−GAT_ values can be influenced by specific systemic and ocular factors. In the high myopia group, univariate linear regression showed that IOP_NCT−GAT_ was positively associated with BMI, AL, and CCT, and negatively associated with CC and IOP_GAT_ (Table 4). Multivariable regression confirmed that IOP_NCT−GAT_ was positively associated with BMI (standardized β = 0.091, *p* = 0.009), SBP (standardized β = 0.074, *p* = 0.027), and CCT (standardized β = 0.506, *p* < 0.001), and negatively associated with age (standardized β = −0.134, *p* < 0.001) and IOP_GAT_ (standardized β = −0.409, *p* < 0.001). The related regression plots (Figure 5) showed that the *R*^2^ values of CCT and IOP_GAT_ on IOP_NCT−GAT_ were 0.167 and 0.069, respectively.

**Table 4:** Univariate and multivariable linear regression analyses of factor that affect IOP_NCT−GAT_.

	**Univariable linear regression**			**Multivariable linear regression**		
	***B*** **(95% CI)**	**β (95% CI)**	***p*** **-value**	***B*** **(95% CI)**	**β (95% CI)**	***p*** **-value**
Age, years	−0.010 (−0.025, 0.006)	−0.045 (−0.118, 0.028)	0.226	−0.029 (−0.042, −0.015)	−0.134 (−0.199, −0.070)	**<0.001**
Sex	−0.141 (−0.458, 0.175)	−0.033 (−0.106, 0.041)	0.381			
BMI (kg/m^2^)	0.063 (0.013, 0.112)	0.092 (0.019, 0.165)	**0.013**	0.062 (0.016, 0.108)	0.091 (0.0.23, 0.159)	**0.009**
SBP (mmHg)	0.009 (−0.002, 0.020)	0.062 (−0.011, 0.135)	0.096	0.011 (0.001, 0.021)	0.074 (0.008, 0.139)	**0.027**
DBP (mmHg)	0.019 (0.003, 0.034)	0.090 (0.017, 0.163)	**0.016**			
AL (mm)	0.231 (0.103, 0.359)	0.131 (0.058, 0.203)	**<0.001**	0.084 (−0.047, 0.214)	0.048 (−0.026, 0.122)	0.207
SE (D)	−0.058 (−0.127, 0.011)	−0.061 (−0.134, 0.012)	0.100			
CCT (μm)	0.027 (0.022, 0.031)	0.407 (0.340, 0.474)	**<0.001**	0.033 (0.029, 0.037)	0.506 (0.442, 0.570)	**<0.001**
CC (D)	−0.206 (−0.312, −0.100)	−0.141 (−0.214, −0.069)	**<0.001**	0.040 (−0.070, 0.149)	0.027 (−0.048, 0.102)	0.474
IOP_GAT_ (mmHg)	−0.227 (−0.287, −0.166)	−0.263 (−0.334, −0.192)	**<0.001**	−0.352 (−0.407, −0.297)	−0.409 (−0.473, −0.345)	**<0.001**

*SBP and DBP, Al and SE were correlated; therefore, only SBP and AL were included in multivariable regression*.

*Boldface values indicate statistical significance*.

*AL, axial length; B, non-standardized beta; BMI, body mass index; β, standardized beta; CC, corneal curvature; CCT, central corneal thickness; CI, confidence interval; D, diopter; DBP, diastolic blood pressure; GAT, Goldmann applanation tonometer; IOP_GAT_, intraocular pressure measured with Goldmann applanation tonometer; IOP_NCT−GAT_, the difference in IOP measured by non-contact and Goldmann applanation tonometer; SBP, systolic blood pressure; SD, standard deviation; SE, spherical equivalence*.

## Discussion

To the best of our knowledge, this is the first study to comprehensively compare the differences between the GAT and NCT measurements and factors that affect them in a non-pathological high myopia population. Among the high myopia participants with a mean age of 31.10 years and IOP of <30 mmHg, although the IOP measurements of the two devices were significantly correlated (*r* = 0.681, *P* < 0.001), IOP_NCT_ overestimated IOP_GAT_ by 3.75 ± 2.10 mmHg. SBP and CCT were the main factors influencing both measurements. Younger age, higher BMI, higher SBP, thicker CCT, and lower IOP_GAT_ significantly broadened the difference in IOP_NCT−GAT_.

In this cross-sectional study, the mean IOP_NCT_ and IOP_GAT_ values were 17.60 and 13.85 mmHg, respectively. Li et al. showed that IOP_GAT_ was 15.1 ± 2.4 mmHg in highly myopic population with a mean age of 22.8 years (14), and a Spanish study suggested that IOP_GAT_ was 15.54 ± 2.78 mmHg in a population with a mean age of 33.8 years (19); these values were slightly higher than those in the present study. Studies regarding IOP_NCT_ in the high myopia population are limited (20). In normal young subjects, IOP_NCT_ usually overestimates IOP_GAT_ by 1–2 mmHg (21–23). Compared with that in our study, the mean difference between IOP_NCT_ and IOP_GAT_ in the present study was greater, which might be related to the different measuring principles and corneal biomechanics in high myopia populations.

The GAT is based on the area of flattened cornea (approximately 7.35 mm^2^), which is converted in mmHg following the Imbert-Fick law (24). The pneumatic system of the NCT generates a puff of air and flattens the central cornea (approximately 10.17 mm^2^), and the time required for applanation is measured and converted to the IOP value (25). We believe that the flattening of the corneal area by the NCT is larger than that by the GAT, and air flattening is more sensitive to ocular surface conditions and corneal structural properties. In our study, the Bland-Altman consistency analysis revealed that the mean difference between the two measurements was 3.75 mmHg, and the 95% upper LOA was 7.86 mmHg. Although there was a correlation between the values obtained by the two devices, their measurement difference was not acceptable from a clinical point of view, as it can affect the correct assessment of IOP, especially in those high myopia patients with a greater probability of combining glaucoma. In general, IOP_NCT_ cannot simply substitute IOP_GAT_ in patients with high myopia.

IOP measurements are affected by various factors. The present study demonstrated that CCT and SBP are the most important factors that can significantly affect IOP_NCT_, IOP_GAT_, and IOP_NCT−GAT_. The effect of CCT was expected, which is in line with most other reports (14, 26–30). Because the slope estimate in NCT is slightly steeper than that in GAT (Figures 3, 4), CCT had a greater influence on IOP_NCT_ than on IOP_GAT_. Thus, it is easy to understand that IOP_NCT−GAT_ increases as the CCT increases. This finding is in good agreement with those of other studies (26, 28, 29).

Blood pressure is another risk factor that has been widely investigated in the context of IOP measurements. Higher SBP may increase aqueous humor drainage by increasing capillary pressure and decreasing outflow by elevating episcleral venous pressure (31). The Japanese Kumejima Study, the European Prospective Investigation into Cancer-Norfolk Eye Study, and the Northern Ireland Cohort for the Longitudinal Study of Aging (NICOLA) reported that higher SBP was a strong determinant of higher IOP (32–34); this finding is consistent with that of the present study. Clinicians should consider the potential effects of CCT and SBP on both types of IOP measurements.

We reported that younger age and higher BMI were significantly correlated with higher IOP_NCT_ but not IOP_GAT_, which is in line with previous studies (13, 14, 31, 32, 35–37). However, the Anyang Childhood Eye Study and NICOLA Study found that IOP increased with older age (34, 38). It has been proposed that both corneal hysteresis and corneal resistance factor values decrease with aging (39). In high myopia populations, corneal hysteresis was reported to be lower than that in normal subjects (40–42). Overall, age-dependent changes in corneal biomechanical properties as well as changes in high myopia may account for our results.

In multivariable analysis, higher CC was a risk factor for increased IOP_GAT_ but not for IOP_NCT_. This result is consistent with that of a previous study (43). Theoretically, a steeper cornea may require greater flattening and deformation to reach a standard contact area; thus, more pressure and higher IOP_GAT_ readings were generated. However, other studies reported that CC affected IOP measurement with dynamic contour tonometry but not with GAT (44, 45). At present, the influence of CC on IOP measurements is inconclusive, and further research is needed to elucidate the reasons for this discordance. Findings on the association between myopia and IOP have also been inconsistent (3, 14, 37, 46). Due to the strong association between AL and SE, and given that SE is affected by more factors than AL, only AL was incorporated into multivariable analysis. However, there was no significant association between AL and IOP in our study; although AL was longer in patients with high myopia, it did not affect the IOP readings. Differences in mechanical strain of the sclera and organizational compliance may explain these findings (37).

Moreover, the present findings suggest that IOP_NVT−GAT_ values increased as IOP_GAT_ values decreased (Table 4), indicating that with the lowering of IOP, the measurement accuracy of IOP_NCT_ decreased. This finding is consistent with that of a previous study, which reported that NCT overestimated IOP in the lower value range and underestimated IOP in the higher value range (22). However, some studies reported opposite findings, suggesting that IOP_NCT_ values were approximately equal to IOP_GAT_ values in the group with low IOP, while they were overestimated in that with high IOP (10, 23). Larger sample sizes are required to validate these results.

Our study has several strengths. First, this was a large cross-sectional study of non-pathologic high myopia patients. Second, the variables of interest included demographic characteristics, AL, SE, CCT, and CC values. Third, we used the NCT and GAT, which are most commonly used in clinical practice, and we examined factors that affect the accuracy of their measurements. The present results highlight the following: (1) CCT and SBP have a strong effect on IOP_NCT_, IOP_GAT_, and IOP_NCT−GAT_ measurements in high myopia patients; (2) younger age and higher BMI values contributed to higher IOP_NCT_ and IOP_NCT−GAT_; (3) CC positively affected IOP_GAT_ measurement; (4) IOP_NCT−GAT_ increased in the higher IOP_GAT_ range; and (5) sex and excessive AL in high myopia patients were not associated with IOP measurements.

There are some limitations to this study. First, this cross-sectional study examined correlations among the relevant factors; however, no causal inferences can be made from the presented findings. Further longitudinal studies are required to confirm these findings. Second, corneal biomechanical properties were not accounted for, including corneal hysteresis and corneal resistance factor, which may greatly affect IOP measurements. Other risk factors, such as smoking and drinking, should also be included in future studies. Third, a control group of age-matched healthy subjects should be included in the analysis. It remains unclear whether the present findings are unique to patients with high myopia. Fourth, the range of IOP_GAT_ in our subjects was 8 to 24 mmHg, and the number of patients with higher IOP values was relatively small. High myopia patients with higher IOP levels may be underrepresented in our sample. Studies involving larger samples with a wide range of IOP values or population-based study are required to provide reliable evidence on IOP measurements obtained with different methods in patients with high myopia.

In conclusion, we believe that IOP_NCT_ cannot simply substitute IOP_GAT_ in high myopia populations, in particular, in patients with thicker CCT and lower IOP values, which are the main factors that broaden the difference in IOP_NCT−GAT_. Moreover, the difference between IOP_NCT_ and IOP_GAT_ and the factors that affect it, including demographic and ocular characteristics, should be considered when evaluating the IOP values. Further studies involving more participants and accounting for corneal biomechanical properties are needed to determine the reliability of different IOP measurements in highly myopic patients.

## Data Availability Statement

The original contributions presented in the study are included in the article/supplementary material, further inquiries can be directed to the corresponding authors.

## Ethics Statement

The studies involving human participants were reviewed and approved by the Ethics Review Committee of the Zhongshan Ophthalmic Center, Sun Yat-sen University, Guangzhou. The patients/participants provided their written informed consent to participate in this study.

## Author Contributions

PW: design and data screening and manuscript drafting. PW, YS, FL, XG, and WC: acquisition, analysis, and interpretation of data. ZW: statistical analysis. MC, YP, and YL: collected and measured data. XZ and SC: study concept and design, project supervision, and manuscript revision. All authors discussed the results and approved the submitted version.

## Funding

This study was supported by the High-level Hospital Construction Project, Zhongshan Ophthalmic Center, Sun Yat-sen University (303020104) and the Science and Technology Program of Guangzhou, China (202102010209).

## Conflict of Interest

The authors declare that the research was conducted in the absence of any commercial or financial relationships that could be construed as a potential conflict of interest.

## Publisher's Note

All claims expressed in this article are solely those of the authors and do not necessarily represent those of their affiliated organizations, or those of the publisher, the editors and the reviewers. Any product that may be evaluated in this article, or claim that may be made by its manufacturer, is not guaranteed or endorsed by the publisher.
